# Effects of traditional Chinese medicine on outcomes and costs of dementia care: results from a retrospective real-world study

**DOI:** 10.1007/s40520-024-02858-9

**Published:** 2024-10-12

**Authors:** Yi-Xiang Weng, Chien-Chung Yang, Wen-Chuin Hsu, Raymond N. Kuo

**Affiliations:** 1 Department of Traditional Chinese Medicine, Chang Gung Memorial Hospital, Taoyuan, Taiwan; 2 School of Traditional Chinese Medicine, College of Medicine, Chang Gung University, Taoyuan, Taiwan; 3 Dementia Center, Department of Neurology, Linkou Chang Gung Memorial Hospital, Taoyuan, Taiwan; 4 School of Medicine, College of Medicine, Chang Gung University, Taoyuan, Taiwan; 5 Institute of Health Policy and Management, College of Public Health, National Taiwan University, Room 632, No.17, Syujhou Rd., Taipei City, 100 Taiwan; 6 Population Health Research Center, College of Public Health, National Taiwan University, Taipei, Taiwan

**Keywords:** Dementia, Traditional Chinese medicine, Clinical dementia rating (CDR), Mini-mental status examination (MMSE), Medical expenses

## Abstract

**Objectives:**

This study aims to assess the impact of Traditional Chinese Medicine (TCM) on dementia patients, utilizing real-world data. Specifically, it seeks to evaluate how TCM influences clinical outcomes by examining changes in the Clinical Dementia Rating (CDR) and Mini-Mental State Examination (MMSE) scores, as well as its effect on medical expenses over a two-year period. Data from a multi-center research database spanning from 2004 to 2021 will be used to achieve these objectives, addressing the current gap in empirical data concerning intuitive outcomes and cognitive function assessments.

**Methods:**

Propensity score matching was adopted to improve comparability among the intervention and control groups. Due to repeated dependent variable measurements, the generalized estimating equation was used to control for socio-demographic characteristics, regional characteristics, and Western medicine treatments for dementia.

**Results:**

After propensity score matching, a total of 441 research subjects were included: 90 in the TCM intervention group and 351 in the non-TCM intervention group. The results of multivariate regression analysis showed that compared with the non-TCM intervention group, the MMSE scores in the TCM intervention group increased by 0.608 points each year. The annual change in CDR scores in the TCM intervention group was 0.702 times that of the non-TCM utilization group. After TCM intervention, annual outpatient expenses increased by US$492.2, hospitalization expenses increased by US$324.3, and total medical expenses increased by US$815.9, compared with the non-intervention group.

**Conclusions:**

TCM interventions significantly decelerate cognitive decline in dementia patients, evidenced by slower reductions in MMSE scores and mitigated increases in CDR scores. However, these benefits are accompanied by increased medical expenses, particularly for outpatient care. Future healthcare strategies should balance the cognitive benefits of TCM with its economic impact, advocating for its inclusion in dementia care protocols.

**Supplementary Information:**

The online version contains supplementary material available at 10.1007/s40520-024-02858-9.

## Introduction

The prevalence of dementia has emerged as a major public health concern due to the growing elderly population across the globe. According to estimates from 2015, approximately 46.8 million individuals were living with dementia worldwide, with the total cost placed at US$818 billion [1], and a further 9.9 million people would be diagnosed every year. By 2030, it is predicted that the number of people living with dementia will reach 75 million. Thus, dementia will persist as a heavy burden for families and society, highlighting the urgent need for effective interventions to address the escalating burden of this debilitating condition.

Although acetylcholinesterase inhibitors and N-methyl-D-aspartate receptor antagonists exhibit efficacy for Alzheimer’s disease during the first year of treatment, they have not been shown to cure nor halt the progression of dementia in long-term follow-ups [2–4]. Many patients also refuse this treatment due to gastrointestinal side effects such as nausea and vomiting [5].

TCM is a complementary therapy that includes Chinese medicine, acupuncture, moxibustion, and tuina massage. Chinese medicine presents a offers a multifaceted therapeutic strategy for dementia patients by modulating chronic inflammatory pathways, attenuating oxidative stress, regulating the ubiquitin-proteasome system, and promoting autophagy [6]. Previous studies have found that acupuncture can improve cognitive function and MMSE scores in patients with Alzheimer’s disease and vascular dementia [7–9]. In clinical trials and a last systematic review, TCM has been shown to improve cognitive functioning, neuropsychiatric symptoms, agitation, and activities of daily living in patients with dementia [10–13]. Nonetheless, these investigations lack long-term follow-ups, especially critical in chronic diseases such as dementia.

Past research utilizing secondary data has revealed that TCM can reduce not only the incidence and the risk of mortality of dementia, aspiration, and pneumonia but also the need for urinary catheters, nasogastric tubes, and tracheostomy tubes [14–17]. However, these studies did not include cognitive performance data and daily functioning as outcomes. MMSE and CDR are essential and widely used measures for assessing the prognosis of dementia. These tools play a critical role in clinical practice, aiding healthcare professionals in diagnosing and tracking the progression of dementia in patients [18].

This study combined CDR and diagnostic codes to screen dementia patients more accurately. In Taiwan, with TCM covered by the National Health Insurance and minimal financial burden on the population. To our knowledge, this study is the first to explore the long-term effects of TCM therapy on MMSE scores, CDR, and medical expenses based on real-world data.

## Method

### Data source

The subjects in this study comprised patients with dementia in the CGRD from 2004 to 2021. We extracted the patients’ CDR, MMSE, and costs in the two years following the initial CDR report. The CGRD is an electronic medical record database from multiple hospitals in a single healthcare system. The CGRD represents approximately 10% of the inpatient and outpatient data from Taiwan’s National Health Insurance Research Database(NHIRD). The CGRD provides more comprehensive clinical data, including MMSE and CDR, compared to the NHIRD [19, 20]. The database includes patients’ medical records at the Keelung, Linkou, Taipei, Taoyuan, Chiayi, Yunlin, Fengshan, and Kaohsiung branches of Chang Gung Memorial Hospital.

## Study population

The study sample included patients who had been assigned the following diagnostic codes at least twice within a year. The diagnostic codes used in this study were based on the International Classification of Diseases, Ninth and Tenth Revision, Clinical Modification (ICD-9-CM and ICD-10-CM), and included codes commonly associated with dementia and related conditions (Appendix 1), such as 290, 294, 331, F01, F02, F03, G30, and G31 [21, 22]. The study start date for each patient was the date of CDR report, where they scored 1 or 2. Patients were required to have both CDR and MMSE scores for two consecutive years after the initial assessment.

The experimental group consisted of patients who received TCM treatments, including acupuncture and herbal medicine, at least six times within the first year of inclusion and another six times during the following year. The control group included patients who did not receive any TCM treatments in the year before their inclusion or during the follow-up period.

Exclusion criteria included patients diagnosed with mild cognitive impairment (MCI), those with a CDR score below 1 or above 2, those without CDR or MMSE scores for two consecutive years, and those lacking socio-demographic data.

## Control variables

The control variables included (1) socio-demographic characteristics such as gender, age, educational background, hypertension, hyperlipidemia, and comorbidity index, (2) regional characteristics such as the hospital branch where they sought medical attention and the degree of urbanization of residence, and (3) whether they received Western medication for dementia. Educational background was a categorical variable in which the patients were divided into two groups: those who received more than nine years of education and those who received nine or fewer years of education. For the comorbidity index, we used the Deyo-Charlson comorbidity index defined by Quan et al., which is determined using codes ICD-9-CM and ICD-10-CM [23]. For hypertension, we searched for ICD9-CM codes 401–405 or ICD10-CM codes I10-I15 and divided the patients into two groups. For hyperlipidemia, we searched for ICD9-CM codes 272.0-272.2 or ICD10CM codes E78.0-E78.2 and also divided the patients into two groups. For the degree of urbanization of residence, which was a categorical variable, we referred to the study conducted by Liu et al., which divided Taiwan’s urban and rural areas into seven degrees of urbanization [24] before dividing the patients into two groups. Finally, we also divided the patients into two groups based on whether they took acetylcholinesterase inhibitors (including donepezil, rivastigmine, and galantamine) and N-methyl-D-aspartate receptor antagonists (including memantine).

### Statistical analysis

To address potential differences in demographic characteristics and other personal conditions between the experimental and control groups, propensity score matching (PSM) was applied. We matched participants based on control variables and severity of dementia. This approach was taken to mitigate selection bias and account for factors influencing TCM utilization in patients with dementia. For categorical variables, exact matching was implemented to enhance intergroup distribution consistency and reduce selection bias. We employed a 1:4 propensity score matching strategy, utilizing greedy matching with a caliper width of 0.2.

We utilized a methodology known as ‘difference-in-differences’ (DID) to perform a comparative analysis of the variances between two adjacent assessments of the CDR and MMSE scores. The objective was to examine and contrast the modifications in the report scores meticulously.

Given the repeated measurements in the CDR, MMSE scores and medical expenses, we employed generalized estimating equation(GEE) models(SAS PROC GENMOD) with a logit link function and an exchangeable correlation structure. This methodology enabled us to account for control variebles, facilitating the examination of the influence of TCM utilization.

Due to the ceiling and floor effects and curvilinear nature of scale scores, metric properties must be calibrated to standardize the distributions of the scale scores [25]. The influence of TCM interventions on the changes in MMSE scores for two consecutive years was determined using linear regression [26, 27]. Linear regression analysis also was conducted to assess the medical expenses. The influence of TCM interventions on the changes in CDR scores for two consecutive years was determined using multinomial logistic regression analysis. SAS9.4 was used to perform our analyses, with p-value < 0.05 as the criterion for statistical significance.

## Result

**Fig. 1:**
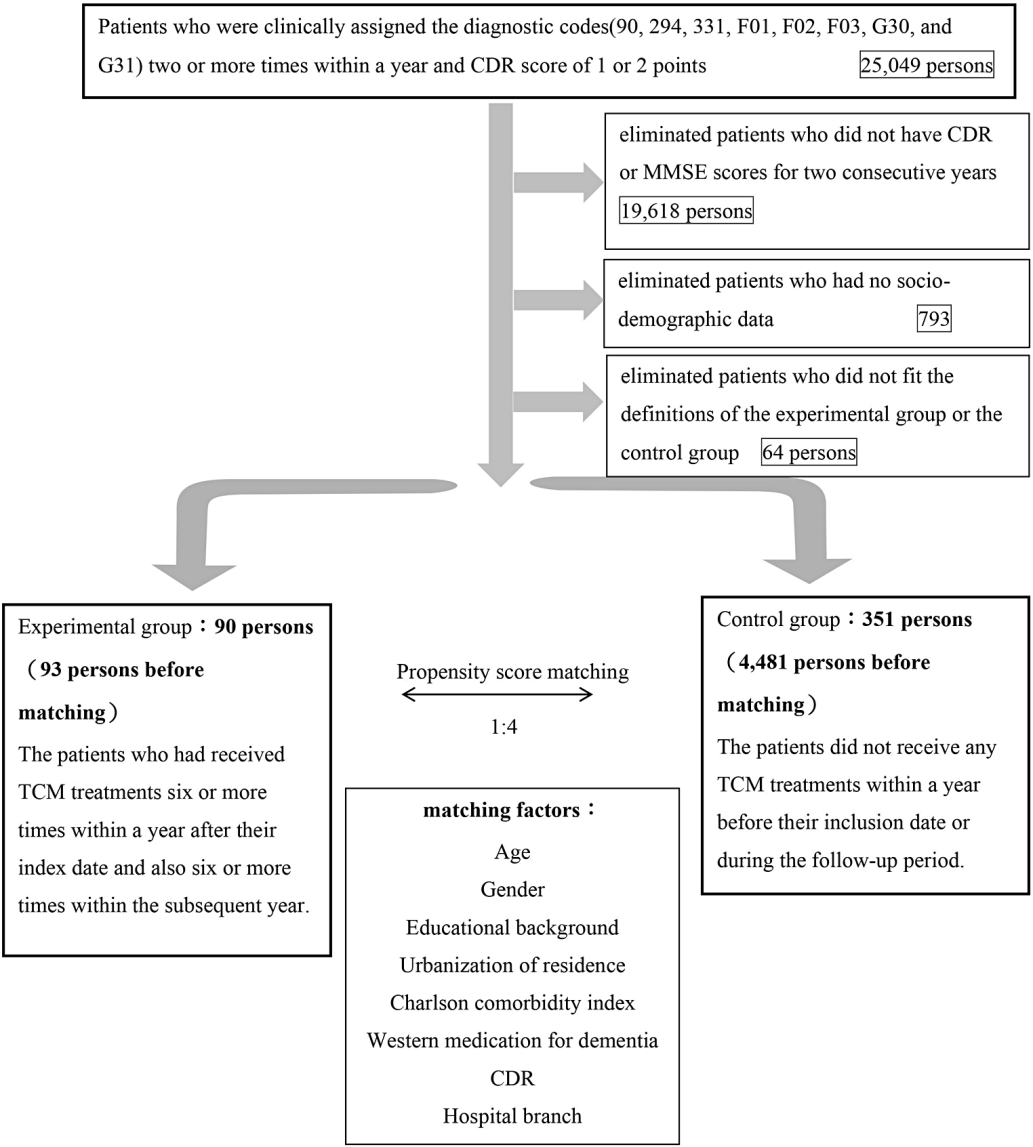
A Flowchart of subject recruitment from Chang Gung Medical Research Database(CGRD) from 2004 to 2021 in Taiwan

We collected data on patients who had a CDR of 1 or 2 and were clinically assigned the aforementioned diagnostic codes two or more times within a year (25,049 patients). After eliminating patients who did not have CDR or MMSE scores for two consecutive years (19,618 patients), had no socio-demographic data (793 patients), and did not fit the definitions of the experimental group or the control group (64 patients), we obtained a total of 4,547 patients, including 93 patients in the experimental group and 4,481 patients in the control group (Table 1). Using a matching ratio of 1:4, we assigned 90 patients to the experimental group and 351 patients to the control group (Fig. 1) (Table 2).

**Table 1:** Demographic and medical characteristics before propensity score matching

variables	TCM user (*N* = 93)		Non-TCM user (*N* = 4,481)		*P*-value
	*n*	(%)	*n*	(%)	
**Age**(Mean, SD)	75.22	8.85	77.02	8.67	**0.047**
**Gender**					0.465
Male	36	38.7	1,571	35.1	
Female	57	61.3	2,910	64.9	
**Educational background**					**< 0.001**
Nine or fewer years	59	63.4	3,566	79.6	
More than nine years	34	36.6	915	20.4	
**Hospital branch**					0.074
Keelung	0	0	3	0.1	
Linkou	18	19.4	1,305	29.1	
Taoyuan	12	12.9	759	16.9	
Chiayi	7	7.5	418	9.3	
Kaohsiung	54	58.1	1,837	41.0	
Yunlin	0	0	32	0.7	
Fengshan	2	2.2	127	2.8	
**Western medication for dementia**					0.539
No	8	8.6	474	10.6	
Yes	85	91.4	4,007	89.4	
**Urbanization**					**0.042**
Rural areas	13	14.0	1,027	22.9	
Urban areas	80	86.0	3,454	77.1	
**CCI** (Mean, SD)	1.75	1.93	0.96	1.37	**< 0.001**
**Hypertension**					0.678
No	51	55.0	2,554	57.0	
Yes	42	45.0	1,927	43.0	
**Hyperlipidemia**					0.053
No	74	79.6	3,877	86.5	
Yes	19	20.4	604	13.5	
**initial CDR**					0.543
CDR = 1	76	81.7	3,546	79.1	
CDR = 2	17	18.3	935	20.9	
**Initial MMSE**(Mean, SD)	16.17	5.53	14.28	5.12	**< 0.001**

**Table 2:** Demographic and medical characteristics after propensity score matching

variables	TCM user (*N* = 90)		Non-TCM user (*N* = 351)		*P*-value
	*n*	(%)	*n*	(%)	
**Age**(Mean, SD)	75.62	8.63	75.11	9.73	0.652
**Gender**					0.857
Male	34	37.8	129	36.8	
Female	56	62.2	222	63.3	
**Educational background**					0.928
Nine or fewer years	58	64.4	228	65.0	
More than nine years	32	35.6	123	35.0	
**Hospital branch**					1.000
Linkou	18	20.0	72	20.5	
Taoyuan	11	12.2	44	12.5	
Chiayi	7	7.8	28	8.0	
Kaohsiung	52	57.8	199	56.7	
Fengshan	2	2.2	8	2.3	
**Western medication for dementia**					0.978
No	7	7.8	27	7.7	
Yes	83	92.2	324	92.3	
**Urbanization**					0.906
Rural areas	13	14.4	49	14.0	
Urban areas	77	85.6	302	86.0	
**CCI** (Mean, SD)	1.61	1.77	1.46	1.67	0.438
**Hypertension**					0.660
No	49	54.4	182	51.9	
Yes	41	45.6	169	48.2	
**Hyperlipidemia**					0.621
No	71	78.9	285	81.2	
Yes	19	21.1	66	18.8	
**Initial CDR**					0.827
CDR = 1	74	82.2	292	83.2	
CDR = 2	16	17.8	59	16.8	
**Initial MMSE**(Mean, SD)	16.22	5.57	15.39	4.88	0.160

The majority of both groups had an initial CDR of 1. In the TCM group, 74 patients had an initial CDR of 1 (82.2%), and 16 patients had an initial CDR of 2 (17.8%). In the non-TCM group, 292 patients had an initial CDR of 1 (83.2%), and 59 patients had an initial CDR of 2 (16.8%). A chi-square test revealed no significant differences between the two groups (*p* = 0.827).

The mean initial MMSE score of the TCM and non-TCM groups was respectively 16.22 and 15.39. An independent sample t-test indicated no significant differences between the initial MMSE scores of the two matched groups (*p* = 0.160).

The time between the first, second, and third reports for each patient included in this study was approximately one year. The mean interval between the first and second reports was 393.69 days, with a standard deviation of 74.79 days and a median of 375 days. The mean interval between the first and third reports was 773.50 days, with a standard deviation of 90.05 days and a median of 755 days.

After controlling for other variables,, the yearly difference of MMSE scores in the TCM group was significantly greater than in the non-TCM group, increasing by 0.608 points per year (95%C.I.: 0.043 to 1.173, p-value: 0.035) (Table 3). The yearly difference in the CDR of the TCM group was 0.702 times that of the yearly difference in the CDR of the non-TCM group (95%C.I.: 0.499 to 0.989, p-value: 0.043), which was significant (Table 4). In patients with dementia, the natural course of the disease typically results in a gradual annual decline in MMSE scores, while CDR scores progressively increase over time, reflecting the worsening cognitive impairment and functional decline associated with the disease [28–31]. This indicates that, in terms of cognitive function, dementia patients in the TCM group demonstrated a slower annual decline in MMSE scores, with an additional improvement of 0.6 points per year compared to those not using TCM. As the CDR assesses both cognitive function and daily living abilities, a smaller CDR difference indicates a slower progression of dementia. In this study, the TCM group exhibited a 0.7-times slower progression of clinical dementia compared to those not using TCM, further supporting the conclusion that the dementia progression was delayed in the TCM group. Furthermore, the MMSE scores of the patients who had taken Western medication for dementia within a year significantly decreased by 1.566 per year when compared to those of the patients who had not taken any Western medication for dementia (95%C.I.: -2.669 to -0.463, p-value: 0.005). This may be attributed to the fact that the the groups with and without Western medication for dementia represent different types of dementia patients. Those prescribed Western medication are predominantly patients with Alzheimer’s disease, characterized by a continuous decline in cognitive function over time [32]. In contrast, other types of dementia, such as vascular dementia, are typically not treated with Western medication. Patients with vascular dementia often experience a stepwise decline in cognitive function rather than a gradual deterioration [33].

**Table 3:** Multivariable linear regression of TCM for yearly MMSE difference

variables	estimated value	95% confidence interval		*P*-value
**TCM** (ref: non-TCM)	0.608	0.043	1.173	**0.035**
**Western medication for dementia** (ref: no use)	-1.566	-2.669	-0.463	**0.005**
**Gender** (ref: female)	-0.529	-1.078	0.021	0.059
**Age**	0.020	-0.015	0.055	0.263
**Hospital branch** (ref: Kaohsiung)				
Linkou	-0.088	-0.671	0.496	0.768
Taoyuan	-0.251	-1.039	0.537	0.532
Chiayi	0.355	-0.381	1.091	0.345
Fengshan	0.887	0.079	1.695	**0.032**
**Educational background** (ref: nine or fewer years)	-0.170	-0.764	0.423	0.574
**Urbanization** (ref: rural areas)	-0.534	-1.215	0.146	0.124
**Hypertension** (ref: no hypertension)	0.518	-0.006	1.042	0.053
**Hyperlipidemia** (ref: no hyperlipidemia)	-0.276	-0.929	0.378	0.408
**CCI**	0.018	-0.133	0.170	0.813

**Table 4:** Multivariable multinomial logistic regression analysis of TCM for yearly CDR difference

Variables	Estimated value	95% confidence interval		*P*-value
**TCM** (ref: non-TCM)	-0.354	-0.696	-0.012	**0.043**
**Western medication for dementia** (ref: no use)	0.222	-0.381	0.824	0.471
**Gender** (ref: female)	-0.033	-0.337	0.270	0.830
**Age**	-0.005	-0.020	0.011	0.567
**Hospital branch** (ref: Kaohsiung)				
Linkou	-0.057	-0.423	0.310	0.762
Taoyuan	-0.180	-0.627	0.267	0.429
Chiayi	0.125	-0.427	0.676	0.657
Fengshan	-0.141	-0.675	0.393	0.605
**Educational background** (ref: nine or fewer years of education)	0.274	-0.048	0.597	0.095
**Urbanization** (ref: rural areas)	0.304	-0.173	0.781	0.212
**Hypertension** (ref: no hypertension)	0.201	-0.100	0.502	0.191
**Hyperlipidemia** (ref: no hyperlipidemia)	0.053	-0.290	0.395	0.764
**CCI**	-0.097	-0.186	-0.007	**0.034**

*Note* Odds ratio(OR) of yearly difference in the CDR of the TCM group is 0.702 when compared to those of the non-TCM group, 95% confidence interval: 0.499∼0.989, P-value:0.043

With the other variables controlled, the outpatient medical expenses per year of the TCM group were US$492.2 (exchange rate: 30 NTD to 1 US dollar) higher than those of the non-TCM group (95%C.I.: 267.4 to 717.0, p-value: <0.001), which is significant. The emergency medical expenses of the TCM group per year were US$2.97 less than those of the non-TCM group; however, this was not significant. The hospitalization expenses of the TCM group per year were US$324.3 higher than those of the non-TCM group (95%C.I.: 19.2 to 629.4, p-value: 0.037), which was significant. Thus, the total medical expenses of the TCM group per year were US$815.9 higher than those of the non-TCM group (95%C.I.: 382.8 to 1249.0, p-value: <0.001), which was significant. The outpatient medical expenses per year of the patients who had taken Western medication for dementia were US$490.3 higher than those of the patients who had not taken Western medication for dementia (95%C.I.: 223.2 to 757.3, p-value: <0.001), which was significant. The total medical expenses per year of the patients who had taken Western medication for dementia were US$497.6 higher than those who had not taken Western medication for dementia (95%C.I.: 28.4 to 966.7, p-value: 0.038), which was significant (Table 5).

**Table 5:** Multivariable linear regression of yearly medical expenses

Variables	Estimated value(US $)			
	Outpatient	Emergency	Hospitalization	Total
**TCM** (ref: non-TCM)	**492.2	-3.0	*324.3	**815.9
**Western medication for dementia** (ref: no use)	**490.3	-13.0	18.6	*497.6
**Gender** (ref: female)	-18.3	-4.0	67.0	44.6
**Age**	-3.6	0.0	4.6	1.1
**Hospital branch** (ref: Kaohsiung)				
Linkou	**-344.2	-6.7	**-291.8	**-640.8
Taoyuan	-198.8	7.5	167.1	-25.8
Chiayi	-15.1	1.7	-61.7	-75.0
Fengshan	-258.3	33.0	182.6	-44.1
**Educational background** (ref: nine or fewer years of education)	-18.3	0.5	157.5	140.4
**Urbanization** (ref: rural areas)	19.0	2.9	67.1	90.6
**Hypertension** (ref: no hypertension)	-60.7	*-9.9	-133.5	-203.0
**Hyperlipidemia** (ref: no hyperlipidemia)	92.4	-3.4	-70.3	19.2
**CCI**	23.0	*3.8	*75.6	102.1

*Note* NT$=new Taiwan dollars, of which 1 US $=30 NT$, TCM = traditional Chinese medicine

* P-value < 0.05 ** P-value < 0.001

## Discussion

To the best of our knowledge, this is the first study to use real-world data to investigate the long-term effects of TCM on the outcome of patients with dementia in terms of MMSE and CDR. The results reveal that TCM intervention could slow down the progression of dementia. Multivariate regression analysis showed that compared with the non-TCM intervention group, the MMSE scores in the TCM intervention group increased by 0.608 points each year. The annual change in CDR scores in the TCM intervention group was 0.702 times that of the non-TCM utilization group. Both of these differences were statistically significant. More remarkable changes in MMSE scores indicate that TCM utilization delays the progression of dementia, whereas more minor incremental changes in CDR mean slower dementia progression.

Although similar observational studies are limited, systematic reviews and meta-analyses have yielded comparable findings. Zhang et al. demonstrated that TCM can enhance MMSE scores and activities of daily living in patients with Alzheimer’s disease [11]. However, the longest follow-up in their randomized controlled trial was only 24 weeks, thus precluding an evaluation of the long-term effects of TCM interventions. Wang et al. reported that acupuncture positively affects MMSE scores and activities of daily living in the short term (less than eight weeks) and mid-term (9–12 weeks) for Alzheimer’s patients. Their randomized controlled trial found no significant differences in short-term Clinical Dementia Rating (CDR) scores between acupuncture and acetylcholinesterase inhibitors, and they noted a lack of research on mid- or long-term CDR outcomes [9]. Given that dementia is a chronic condition with an average survival of 8–10 years, this is a critical consideration.

Burback et al. observed that during the natural course of Alzheimer’s disease, the MMSE scores of the patients decreased by an average of 2.8 points per year (95%C.I.: 1.8 to 4.2) [31]. The results of the current study indicate that the second and third MMSE scores of the TCM group were respectively 0.21 and 1.489 points lower than the initial scores, whereas the second and third MMSE scores of the non-TCM group were respectively 0.92 and 2.687 points lower than the initial scores. In another study, May et al. observed that TCM was as effective as donepezil in treating Alzheimer’s disease. Both TCM and donepezil improved the MMSE scores of the patients within a one-year fellow-up: compared to initial scores, the MMSE scores of the TCM group were 1.64 points higher (95%C.I.=1.03 to 2.25), and those of the donepezil group were 1.40 points higher (95%C.I.=0.39 to 2.41). At week 64, the scores of both groups were lower than the initial scores and presented no significant differences [34]. In a one-year randomized controlled trial included in the study conducted by May et al., the MMSE scores increased a year after the start of treatment because the patients in said randomized controlled trial had never previously received treatment. In contrast, the current study was an observational study which included patients who had already received some form of treatment, thereby explaining the decline in MMSE scores. Arai et al. similarly observed that patients already receiving donepezil experienced a decline in MMSE scores of about 1 point over one year and approximately 2 points over two years [3].

Our study employed a difference-in-differences approach with MMSE and CDR to assess the efficacy of TCM in the treatment of dementia. Similar to our study, May et al. utilized the same approach [34], recognizing that monitoring changes in scores among dementia patients over time is standard in clinical practice. Additionally, Taiwan’s National Health Insurance requires annual follow-ups on CDR and MMSE scores to authorize payments for acetylcholinesterase inhibitors and NMDA receptor antagonists used to treat dementia.

Compared to previous observational studies utilizing data from the NHIRD [15–17, 35–38], our research uses diagnostic codes and CDR as inclusion criteria, effectively reducing selection bias. Chern et al. noted that the NHIRD they used lacked the best clinical staging index, namely, the CDR score, which they cited as a limitation [36]. Relying solely on diagnostic codes for inclusion criteria in dementia studies using secondary data can mistakenly include patients with mild cognitive impairment (MCI) [39], leading to selection bias and misinterpretation. Lee et al. further found that using secondary data from Medicare in the US could accurately identify only 85% of dementia patients and 77% of individuals with normal cognitive functions [40]. Therefore, employing CDR or MMSE scores as screening criteria improves the alignment of identified patients with the true definition of dementia [21]. Combining diagnostic codes with cognitive function tests enhances the accuracy of dementia patient identification [22].

Lin et al. found that 43% of dementia patients in Taiwan used TCM [37]. In their study, any patient who visited a TCM clinic within the preceding ten years was classified in the TCM group, introducing a potential for selection bias. We believe that at least six visits to TCM within a year are necessary for accurate classification, as a typical course of acupuncture in Taiwan involves a minimum of six sessions, and TCM prescriptions can last up to one month. Before applying propensity score matching in our study, we identified 4,574 dementia patients, of whom only 93 (2%) were placed in the TCM group. The low utilization rates may be due to our shorter observation period of only two years post-inclusion, compared to the ten-year period used by Lin et al. and Chern et al. [36, 37].

Additionally, although most of our participants regularly sought medical care at Chang Gung Memorial Hospital, they may have received TCM treatment at other hospitals, which would not be captured in the database used. These factors likely account for the lower utilization rates observed in our study compared to those reported by Lin et al. and Chern et al. Furthermore, based on the effect of TCM in delaying the progression of dementia, we recommend that future healthcare policies should increase the utilization of TCM in the treatment of dementia.

This study found that the outpatient and total medical expenses per year of the TCM group were, respectively, US$492.2 and US$815.9 higher than those of the non-TCM group. The outpatient medical expenses per year of the patients who had taken Western medication for dementia were US$490.3 higher than those of the patients who had not taken Western medication for dementia, which is close to the additional amount that the patients in the TCM group paid. Patients who use TCM or take dementia medication regularly in the long term have possibly better health awareness, thereby leading to higher medical expenses. A double-blind, randomized controlled trial conducted by Courtney et al. estimated that the medical expenses of Alzheimer’s disease patients taking donepezil as a treatment were GB£498 higher per year than those of the placebo group [4], which is similar to the results of the current study.

However, Lin et al. observed that the hospitalization costs of their TCM group were, on average, US$867.3 (*P* = 0.015) lower per person than those of their non-TCM group [16]. In their study, the hospitalization costs were accumulated for approximately ten years. The patients included in their study were also newly diagnosed with dementia, which perhaps included mild cognitive impairment or very early stages of dementia.

Furthermore, their definition of TCM intervention was less conservative; in other words, any use of TCM within ten years was deemed a TCM intervention. In contrast, we examined the expenses of TCM interventions by year and employed propensity score matching to compare the two groups more accurately. We found that the hospitalization expenses of the TCM group per year were US$324.3 higher than the non-TCM group; however, our hospitalization data were derived solely from Chang Gung Memorial Hospital. Thus, the findings regarding hospitalization fees could benefit from further examination.

## Limitation

The coverage of the CGRD is not as widespread as that of the NHIRD; therefore, its generalizability to the entire population is limited. And the results may not be generalized to patients from different healthcare settings in other countries.

Furthermore, the results may be confounded by the control group’s exposure to TCM outside the healthcare network, it might underestimate the actual effect of TCM. The results show that TCM therapy exerts a positive impact on dementia patients. If patients who had received TCM therapy outside of Chang Gung Memorial Hospital were removed from the control group, we would derive more significant TCM therapy effects. It won’t alter the conclusion of this study.

The calculation of the medical expenses only included those in the Chang Gung Memorial Hospital system. However, the patients included in this study were patients who had done follow-ups and examinations at Chang Gung Memorial Hospital for an extended period. The possibility that medical expenses incurred outside of Chang Gung Memorial Hospital cannot be excluded.

Finally, dementia treatments can be medication or non-medication; however, non-medication data were not included in the database used in this study. Although this study did not include non-medication treatments as control variables in the regression models, we have matched a wide range of factors, including education, hospital branch, and the level of urbanization of the patient’s residence. The same variables were also controlled in the regression models. These variables might influence the use of non-medication treatments [41].

## Conclusion

TCM interventions have been shown to significantly decelerate the decline in MMSE scores and the progression in CDR scores among patients with dementia. However, TCM interventions are associated with increased medical expenses, particularly in outpatient care. Future healthcare policies aimed at optimizing outcomes for patients with dementia should consider the incorporation of TCM therapies.

## Electronic Supplementary Material

Below is the link to the electronic supplementary material.

Supplementary Material 1

## Data Availability

Data are available from Chang Gung Research Database provided by Chang Gung Memorial Hospital. Due to legal restrictions imposed by the Taiwan government with respect to the “Personal Information Protection Act”, data cannot be made in public. Data requests as a formal proposal can be sent to Chang Gung Memorial Hospital (https://www.cgmh.org.tw/). The interpretation and conclusions contained herein do not represent the position of Chang Gung Memorial Hospital.
