# Supplementary material for: Effects of traditional Chinese medicine on outcomes and costs of dementia care: results from a retrospective real-world study
Source: Aging Clin Exp Res. 2024 Oct 12;36(1):204. doi: 10.1007/s40520-024-02858-9 (PMC11470846; doi:10.1007/s40520-024-02858-9)
Supplement: Supplementary file 1 — Supplementary Material 1 [file 40520_2024_2858_MOESM1_ESM.docx]

**Appendix 1 ICD-9-CM / ICD-10-CMA**

| **ICD-9** | **Disease name and its subclassification** |
| --- | --- |
| 290.0 | Senile dementia |
| 290.1 | Presenile dementia  290.10 Presenile dementia, uncomplicated  290.11 Presenile dementia with delirium  290.12 Presenile dementia with delusional features  290.13 Presenile dementia with depressive features |
| 290.2 | Senile dementia with delusional features  290.20 Senile dementia with delusional features  290.21 Senile dementia with depressive features |
| 290.3 | Senile dementia with delirium |
| 290.40 | Vascular dementia  290.40 Vascular dementia, uncomplicated  290.41 Vascular dementia, with delirium  290.42 Vascular dementia, with delusions  290.43 Vascular dementia, with depressed mood |
| 294.0 | Amnestic disorder in conditions classified elsewhere |
| 294.1 | Dementia in conditions classified elsewhere  294.10 Dementia in conditions classified elsewhere without behavioral disturbance  294.11 Dementia in conditions classified elsewhere with behavioral disturbance |
| 294.2 | Dementia, unspecified  294.20 Dementia, unspecified, without behavioral disturbance  294.21 Dementia, unspecified, with behavioral disturbance |
| 294.8 | Other persistent mental disorders due to conditions classified elsewhere |
| 294.9 | Unspecified persistent mental disorders due to conditions classified elsewhere |
| 331.0 | Alzheimer's disease |
| 331.1 | Frontotemporal dementia  331.11 Pick's disease  331.19 Other frontotemporal dementia |
| 331.2 | Senile degeneration of brain |
| 331.7 | Cerebral degeneration in diseases classified elsewhere |
| 331.82 | Dementia with Lewy bodies |
| 331.89 | Other cerebral degeneration |
| 331.9 | Cerebral degeneration, unspecified |

| **ICD-10** | **Disease name and its subclassification** |
| --- | --- |
| F01.50 | Vascular dementia without behavioral disturbance |
| F01.51 | Vascular dementia with behavioral disturbance |
| F02.80 | Dementia in other diseases classified elsewhere without behavioral disturbance |
| F02.81 | Dementia in other diseases classified elsewhere with behavioral disturbance |
| F03.90 | Unspecified dementia without behavioral disturbance |
| F03.91 | Unspecified dementia with behavioral disturbance |
| G30.0 | Alzheimer's disease with early onset |
| G30.1 | Alzheimer's disease with late onset |
| G30.8 | Other Alzheimer's disease |
| G30.9 | Alzheimer's disease, unspecified |
| G31.01 | Pick's disease |
| G31.09 | Other frontotemporal dementia |
| G31.1 | Senile degeneration of brain, not elsewhere classified |
| G31.83 | Dementia with Lewy bodies |
| G31.85 | Corticobasal degeneration |
| G31.89 | Other specified degenerative diseases of nervous system |
| G31.9 | Degenerative disease of nervous system, unspecified |
